# Indigenous knowledge dataset of Dayak, Malay and Chinese communities in Sintang Regency, West Kalimantan, Indonesia

**DOI:** 10.1016/j.dib.2024.110147

**Published:** 2024-02-06

**Authors:** Adriana Gandasari, Markus Iyus Supiandi, Thomas Joni Verawanto Aristo, Ursula Dwi Oktaviani, Dinn Wahyudin, Asep Herry Hernawan, Anastasia Selvi, Fiony, Mawardi

**Affiliations:** a STKIP Persada Khatulistiwa, Sintang, West Kalimantan, Indonesia; b Universitas Pendidikan Indonesia, Bandung, West Java, Indonesia

**Keywords:** Indigenous ethnicity, Local people, Local wisdom, Local content

## Abstract

The dataset in this article presents indigenous knowledge of Dayak, Malay, and Chinese ethnic communities in Sintang Regency, West Kalimantan, Indonesia. Data was collected by involving three categories of main informants, namely community leaders, the main actors directly involved in indigenous knowledge of their tribe. Key informants are community leaders referred to as leaders or elders in their tribe's indigenous knowledge. They are recommended informants, namely the general Dayak, Malay, and Chinese, who know and maintain indigenous knowledge of their tribes. Research instruments were used to obtain data using open interview sheets, in-depth interviews, and documentation. The data was collected to screen local content owned by Dayak, Malay, and Chinese communities in Sintang Regency, West Kalimantan, Indonesia, that have the value of local wisdom. The urgency of data collection is to document indigenous knowledge to preserve local communities' culture.

Specifications Table

**Table:** 

Subject area	Indigenous Knowledge
Specific subject area	Ethnic Dayak, Ethnic Malay, and Ethnic Chinese at Sintang Regency, West Kalimantan, Indonesia
Data source location	Data collected at fourteen districts at Sintang Regency, West Kalimantan, Indonesia
Type of data	Table, Graph
Data collection	Collected data using open interview sheets, in-depth interviews, and documentation. The method used for collecting data is a survey with twelve scopes: (a) history, (b) language, (c) literature, (d) arts, (e) crafts, (f) customary customs and customary law, (g) technology and tools, (h) natural environment and ecosystem, (i) medicine, (j) cuisine, (k) clothing, and (l) sport. Data was collected using informants of Dayak, Malay, and Chinese consisting of main, key, and recommendation informant.
Data format	Analyzed dataset is available at https://doi.org/10.17632/4byr9p2fv5/1/files/edafd804 and https://doi.org/10.17632/4byr9p2fv5/1/files/2d469504
Data accessibility	Data are included in this article and available at https://doi.org/10.17632/4byr9p2fv5/1

## 1 Value of the Data

• The data set provides coverage of indigenous knowledge of Dayak, Malay, and Chinese ethnic communities in Sintang district, West Kalimantan, Indonesia, which is divided into twelve scopes, namely: (a) history, (b) language, (c) literature, (d) art, (e) crafts, (f) customary customs and law, (g) technology and tools, (h) natural environment and ecosystems, (i) medicines, (j) food and beverages, (k) clothing, and (l) sports.
• Cultural preservation communities can use datasets to realize the preservation of indigenous knowledge of local content with local wisdom value.
• Datasets can be used by the scientific community to aim at understanding and identifying critical points in indigenous knowledge that need special attention.
• The dataset can be used by educators and researchers of social sciences and human/ities related to Dayak, Malay, and Chinese ethnic groups inhabiting the Kalimantan region, especially in Sintang Regency, West Kalimantan, Indonesia, to increase support for the preservation of local content owned.
• Data can be used to find triangular relationships between Dayak, Malay, and Chinese ethnicities about the value of local wisdom in these ethnicities. It gave rise to a unique civilization in each tribe, the role of each tribe as a community of people, and acculturation between the ethnic tribes.

## 2 Data Description

Indigenous knowledge is part of the diversity of cultural values of the Indonesian nation [1], which must be preserved and passed on to the next generation [2] so that it does not experience extinction [3]. Indigenous knowledge needs to be known by the broader community so that mutual respect and respect for each other's cultural values arise [4]. Initial observations made by the research team obtained several things that could threaten indigenous knowledge in Dayak, Malay, and Chinese communities in Sintang Regency who experienced degradation to extinction, namely: (1) Conveyed through traditional media (word of mouth, through myth, and ritual); (2) Changes in perceptions of local communities; (3) Lifestyle changes that are more likely to harm the natural environment; (4) Commercialization and socioeconomic change; and (5) Lack of knowledge and the younger generation's penchant for indigenous knowledge. These initial observations are supported by previous research that identified several problems that threaten the existence of indigenous knowledge, including not being well documented and even not having written documents [5]; orally passed from generation to generation [6]; technological advances and foreign cultures erode local cultures [7]; illegal logging, oil palm plantations [8,9] which cause local endemic flora and fauna to become scarce [10].

The data collected was obtained from 161 informants each of Dayak, Malay and Chinese using purposive sampling consisting of three informants, namely the main informant, who is a Dayak, Malay, Chinese community leader as the leading actor who is directly involved in indigenous knowledge of the tribe; key informants are Dayak, Malay, Chinese community leaders referred to as traditional leaders, community leaders and tribal elders; and recommended informants divided into three categories, namely Dayak, Malay, Chinese who know, who collect and who maintain their tribe's indigenous knowledge. The informant dataset is available at DOI: https://doi.org/10.17632/4byr9p2fv5/1/files/830e818a.

Indigenous knowledge data sources, namely Dayak, Malay, and Chinese, are divided into 12 sections: history, language, literature, arts, crafts, customary customs and customary law, technology and tools, natural environment and ecosystems, medicine, food and beverage, fashion, and sports and divided into variables of which there are at least 105 variables. In the history section, there are 13 subsections; language has 4 subsections; literature has 6 subsections; arts have 5 subsections; crafts have 11 subsections; customary customs and customary law there are 7 subsections; technology and tools there are 10 subsections; natural environment and ecosystems there are 3 subsections, medicine there are 13 subsections, food and beverage there are 11 subsections, fashion there are 18 subsections, and sports there are 4 subsections. The tribal community can utilize this knowledge to maintain and preserve it through local community activities. For example, *Gawai Dayak* is performed by Dayak tribal communities; Malay ethnic communities perform *Kenduri*; and Chinese ethnic communities perform *Cap Go Meh*.

The data code of indigenous knowledge and each variable are available at DOI: https://doi.org/10.17632/4byr9p2fv5/1/files/648b479a. Each variable and category of ease of tracing indigenous knowledge data from the easiest to find to the most difficult to find in percentage form and displayed with dark green, light green, yellow, orange, and red, where these colors represent the level of preservation of indigenous and also indicated the percentage of sustainability (see Table 1).

**Table 1:** Variables in the data file include description, category, and percentage of indigenous knowledge of Dayak, Malay, and Chinese tribes.

Data was collected using open and in-depth interview sheets and documentation. The interview instruments used have been validated based on content, construct and culture. The instrument interview sheet is available at DOI: https://doi.org/10.17632/4byr9p2fv5/1/files/6113f7ba. Open discussions are conducted to allow informants to explain the object of research interviewed so as not to rule out the possibility of new questions arising according to the informant's conversation flow, and in-depth interviews aim to trace informants' answers in as much detail as possible. Researchers prepare 105 main questions according to research variables so that the interview process is open and in-depth interviews stay consistent.

West Kalimantan was formed on 1 January 1957 [11] and is the third largest province with an area of 147,307 km2 [12], equivalent to 7.68 the size of Indonesia, inhabited by three local tribes, namely Dayak [13], Malay [14] and Chinese [15], spread across 14 districts [16]. In addition, there are also other tribes, such as immigrants and mixed tribes. In the Sintang district, this tribe inhabits an area. It develops into a majority tribe with different societal roles [17] and distinctive socio-cultural characteristics [18], namely distinctive indigenous knowledge.

There are 19 Dayak sub-ethnicities in Sintang Regency, namely: Desa, Goneh, Inggar Silat, Kayan, Kebahan, Ketungau, Lebang, Mualang, Nanga, Papak, Paya’, Sebaru, Seberuang, Sekajang, Sekubang, Sekujam, Selawe, Undau, dan Uud Danum. In addition to Dayak sub-ethnicity, each sub-district in Sintang regency, which totals fourteen sub-districts, is also inhabited by Malay tribes so that Malay tribes are identified with the name of the sub-district, namely Ambalau Malay, Binjai Hulu Malay, Dedai Malay, Kayan Hilir Malay, Kayan Hulu Malay, Kelam Permai Malay, Ketungau Hilir Malay, Ketungau Hulu Malay, Ketungau Tengah Malay, Sungai Tebelian Malay, Sepauk Malay, Serawai Malay, Sintang Malay, and Tempunak Malay. In comparison, the Chinese tribe in Sintang Regency consists of three tribes: Hokkian/Hok-lo Chinese, Khek/Hakka Chinese, and Tioceu/Teochew Chinese. The others are migrants and mixtures that in this study were not explored.

**Fig. 1:**
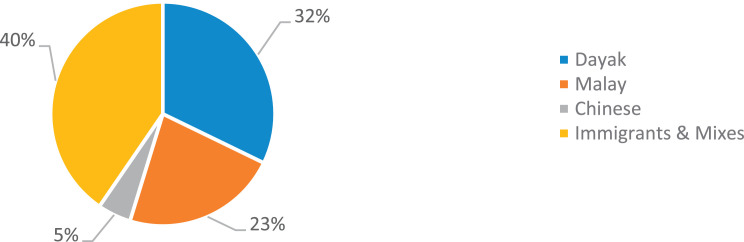
Distribution of tribes in Sintang Regency, West Kalimantan, Indonesia.

The data on ethnic distribution is available at DOI: https://doi.org/10.17632/4byr9p2fv5/1/files/0fc23d9c. The percentage of ethnic distribution in Sintang Regency, West Kalimantan, Indonesia is shown in Fig. 1.

**Fig. 2:**
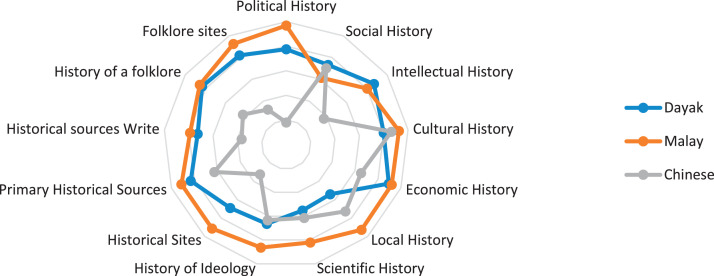
Indigenous knowledge about history.

**Fig. 3:**
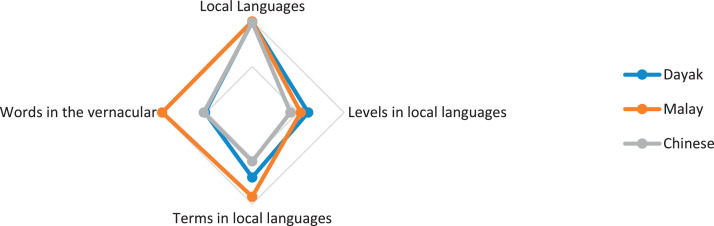
Indigenous knowledge about languages.

**Fig. 4:**
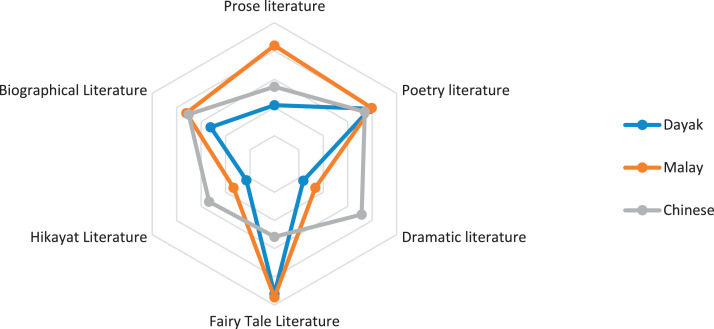
Indigenous knowledge about literature.

**Fig. 5:**
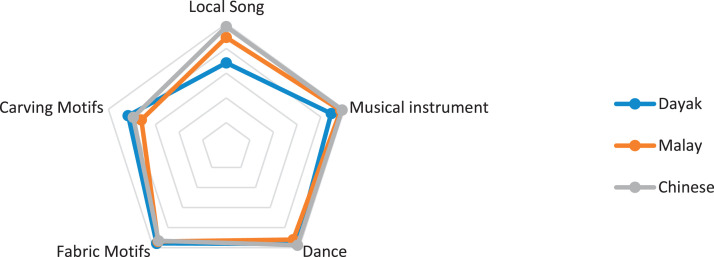
Indigenous knowledge about arts.

**Fig. 6:**
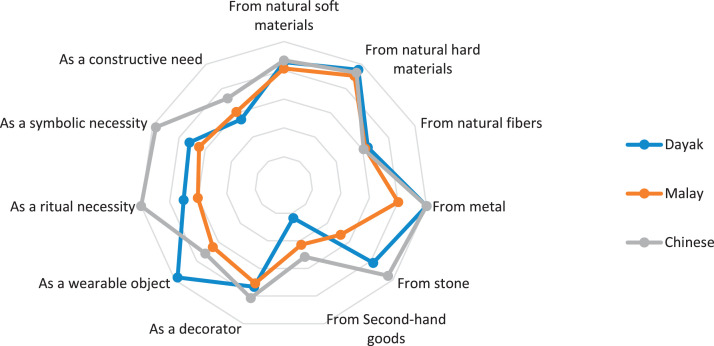
Indigenous knowledge about crafts.

**Fig. 7:**
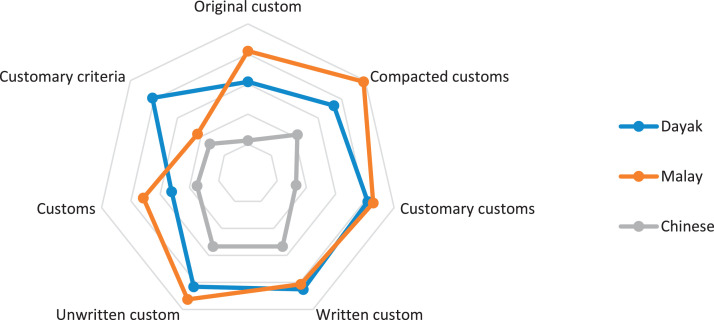
Indigenous knowledge about customary customs and customary law.

**Fig. 8:**
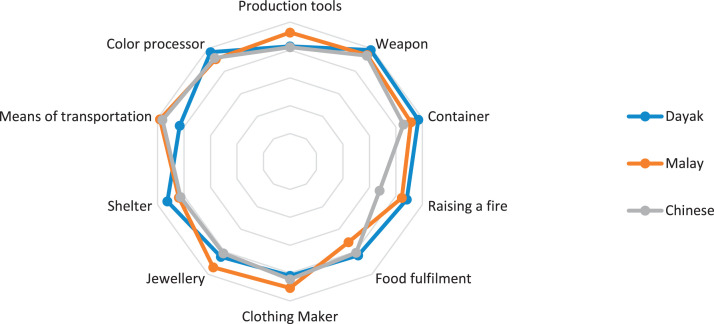
Indigenous knowledge about technology and tools.

**Fig. 9:**
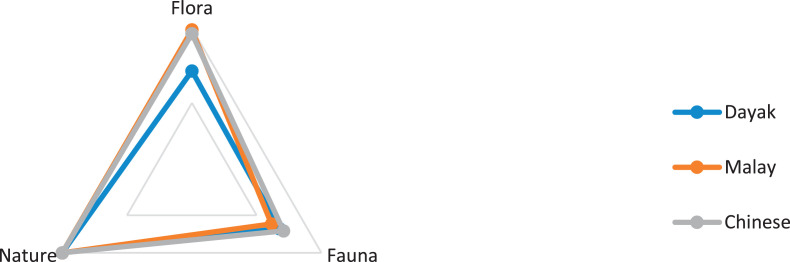
Indigenous knowledge about natural environment and ecosystems.

**Fig.10:**
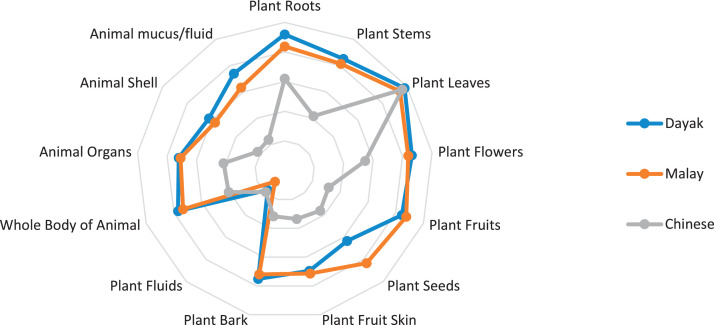
Indigenous knowledge about medicine.

**Fig. 11:**
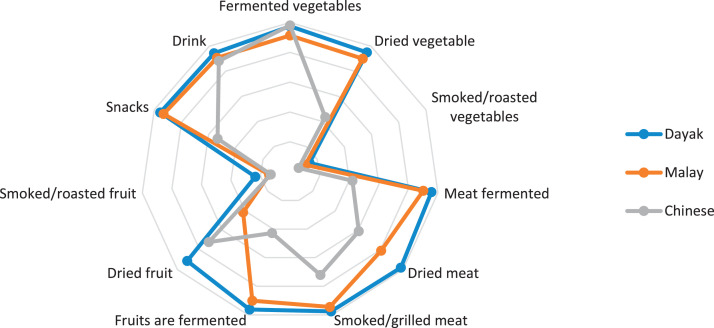
Indigenous knowledge about food and beverages.

**Fig. 12:**
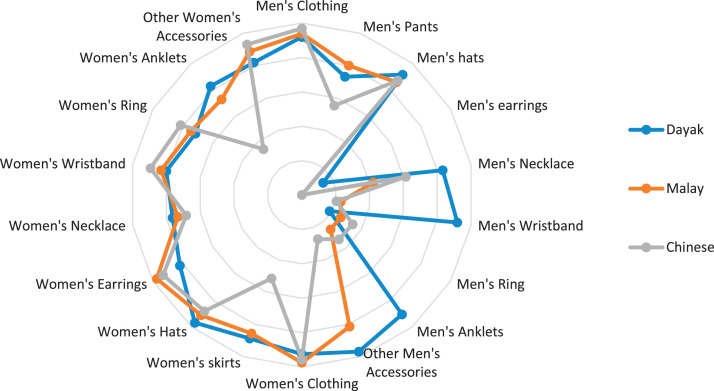
Indigenous knowledge about fashion.

**Fig. 13:**
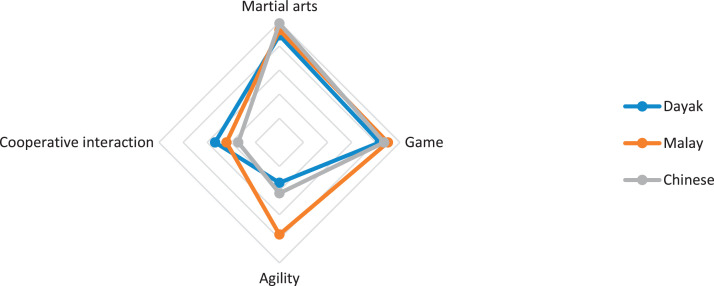
Indigenous knowledge about sports.

This research only focuses on indigenous knowledge data of Sintang Regency, West Kalimantan indigenous people, namely Dayak, Malay, and Chinese communities. The data is shown in the form of a web diagram. The twelve data obtained related to the preservation of indigenous knowledge in Dayak, Malay, and Chinese tribes, namely history (Fig. 2), language (Fig. 3), literature (Fig. 4), arts (Fig. 5), crafts (Fig. 6), customary customs and customary law (Fig. 7), technology and tools (Fig. 8), natural environment and ecosystems (Fig. 9), medicine (Fig. 10), food and beverage (Fig. 11), fashion (Fig. 12), and sports (Fig. 13). The twelve data of indigenous knowledge are available at DOI: https://doi.org/10.17632/4byr9p2fv5/1/files/9a7028cb.

Fig. 2 shows that indigenous knowledge history is dominated by ethnic Malays, namely related to political history. Dayak tribes, indigenous people of Kalimantan islands, are more prominent in economic history data, and Chinese tribes are more pronounced in cultural history. In contrast, the small indigenous knowledge history data is in ethnic Chinese, which is political history, ethnic Dayak, which is local history, and Malay, which is social history.

Fig. 3 shows that local languages, namely Dayak, Malay, and Chinese, are still used by the tribal community, especially in communicating, so this indigenous knowledge data dominates. In contrast, the most minor data are Chinese and Malay tribes related to level in the local language, while Dayak tribes are words in the vernacular.

Fig. 4 shows the dominant indigenous knowledge of literature data in Malay and Dayak tribes, namely in fairy tale literature and Chinese tribes in dramatic literature. In contrast, the lowest data is hikayat literature in the Dayak tribe, in the Malay tribe, namely dramatic and hikayat literature, and in Chinese, namely fairy tale literature.

Fig. 5 shows that indigenous knowledge related to art is still well maintained by being dominated by Chinese tribes; there are three sub-indigenous knowledge, namely in local song, musical instrument, and dance; Malay tribe on musical instrument and Dayak tribe on dance and fabric motifs while the low data are Chinese tribe and Malay tribe on carving motifs and Dayak tribe on local song.

Fig. 6 shows that a ritual necessity dominates indigenous knowledge about craft for the Chinese tribe; the Dayak tribe is more of a wearable object, and the Malay tribe is from natural hard materials; besides that, these three tribes get the smallest value from second-hand goods.

Fig. 7 shows that the dominant indigenous knowledge about customary customs and customary law of the Malay tribe is compacted traditions, Dayak tribe unwritten customs, and Chinese are written businesses. At the same time, those not dominant are the Chinese tribe related to original customs, Malays in customary criteria, and Dayak tribes in customs.

Fig. 8 shows that indigenous knowledge about technology and tools that dominate Dayak tribes is weapons, Chinese and Malay tribes are means of transportation; while the lowest data is on the Chinese tribe related to raising a fire, the Malay tribe is related to food fulfillment and the Dayak tribe is related to clothing makers.

Fig. 9 shows that indigenous knowledge about the natural environment and ecosystems of both Dayak, Malay, and Chinese tribes are related to the tropics because they are in the tropics area, while the lowest data is on Malay, Dayak, and Chinese tribes, namely Endemic fauna.

Fig. 10 shows data on indigenous knowledge about the medicine of Dayak, Chinese, and Malay tribes, namely in plant leaves, while those less used in Malay, Dayak, and Chinese tribes are plant fluids.

Fig. 11 shows that Indigenous Knowledge about Food and Beverages Dayak tribe is more dominant in fermented meat and dried meat, Chinese tribe in fermented vegetables and Malay tribe in smoked/grilled meat; hiwle the data is low on smoked/roasted vegetables in the three tribes.

Fig. 12 shows that indigenous knowledge about fashion in the Malay tribe is dominated by women's clothing and earrings, the Dayak tribe in other men's accessories and women's hats, and the Chinese tribe in women's clothing. In contrast, data is not dominated by Malay, Chinese, and Dayak tribes, namely men's earrings.

Fig. 13 shows that indigenous knowledge about sports in Chinese, Malay, and Dayak tribes is dominant in martial arts, while the lowest data is on cooperative interaction for Malays and Chinese and Dayak tribes on agility.

## 3 Experimental Design, Materials and Methods

**Fig. 14:**
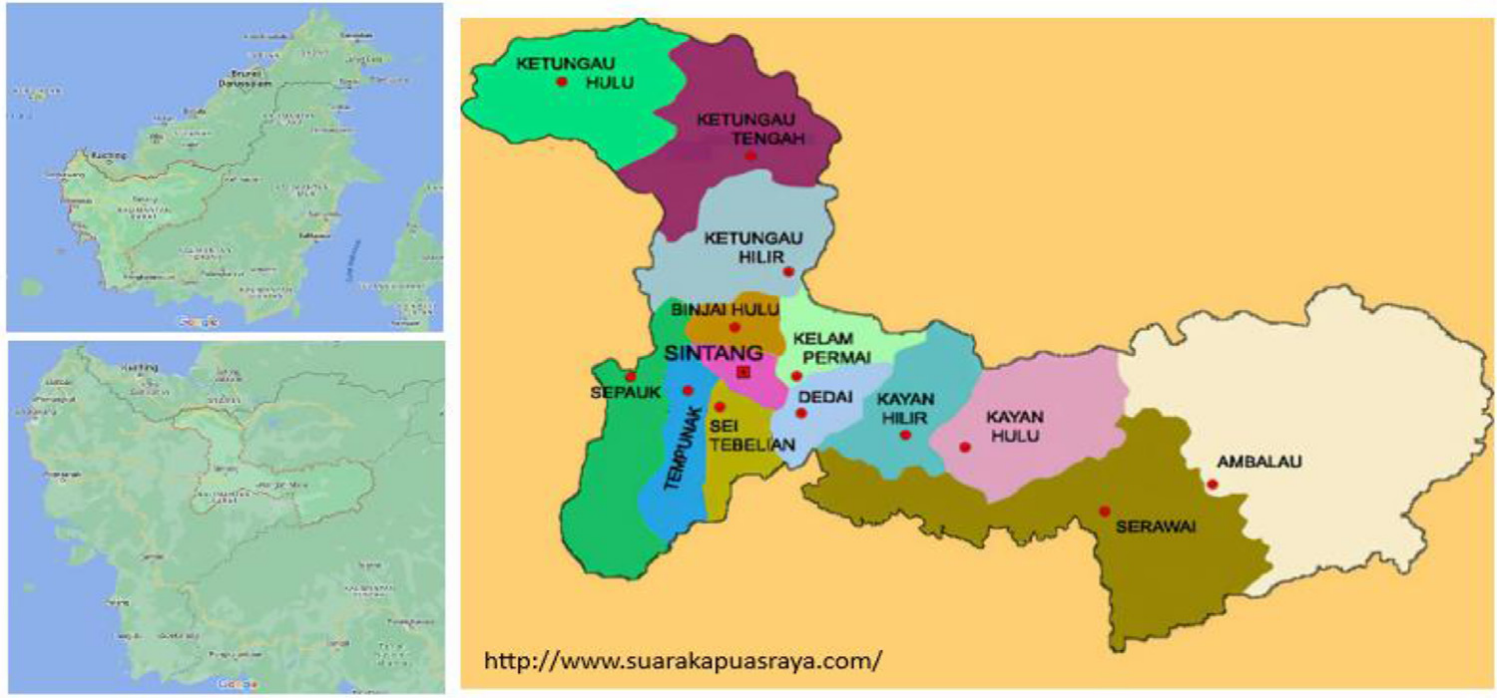
The maps of Sintang Regency where data were collected.

Data collection in the field was carried out in 14 sub-districts in Sintang Regency, namely Serawai, Ambalau, Kayan Hulu, Sepauk, Tempunak, Sungai Tebelian, Sintang, Dedai, Kayan Hilir, Kelam Permai, Binjai Hulu, Ketungau Hilir, Ketungau Tengah and Ketungau Hulu in April-July 2023; it's shown in Fig. 14. Indigenous knowledge data was collected using a survey approach through open interviews and in-depth interviews with key informants, key informants and recommendation informants as well as documentation on the existence of 12 scopes. Open discussions for consultation using 105 main questions with the keyword “*What do you know about …?”* followed by in-depth questions based on answers provided by informants. This profound question leads to two things related to indigenous knowledge: what has been preserved and what has not been maintained. Documentation is carried out during the interview process in written and non-written form.

The informants involved numbered 161 people each Dayak, Malay, and Chinese Ethnic aged 20–70 years, consisting of primary informants, key informants, and recommendation informants. Informants are selected by purposive sampling. They could provide accurate information and had good knowledge of indigenous knowledge of their tribe. The criteria for informants are the main actors, leaders, elders, and figures who know, collect, and maintain indigenous knowledge of their tribe. The existence of informant criteria is intended to obtain accurate data because these informants are qualified in terms of indigenous knowledge of their tribe.

Data collected through questionnaires and interviews are presented as raw data. This data is then analyzed using qualitative descriptive analysis in the form of described percentages. Raw data is available at DOI: https://doi.org/10.17632/4byr9p2fv5/1/files/2d469504 and analysis data is available at DOI: https://doi.org/10.17632/4byr9p2fv5/1/files/edafd804. The results of data analysis are summarized in the form of data recapitulation available at DOI: https://doi.org/10.17632/4byr9p2fv5/1/files/170a91f5 and data on display in the form of tables and graphs available at DOI: https://doi.org/10.17632/4byr9p2fv5/1/files/9a7028cb. All research data are available at DOI: https://doi.org/10.17632/4byr9p2fv5/1.

## Limitations

None or not applicable.

## Ethics Statement

This research was declared ethically appropriate, following 7 (seven) WHO 2011 standards. (1) Social Value, (2) Scientific Value, (3) Equitable assessment and Benefits, (4) Risk, (5) Persuasion Exploitation, (6) Confidentially and Privacy, and (7) Informed Consent, referring to the 2016 CIOMS Guidelines. The fulfillment of the indicator of each standard indicates this. The ethics statement letter is available at https://doi.org/10.17632/4byr9p2fv5/1/files/e1124ea0.

## CRediT authorship contribution statement

**Adriana Gandasari:** Conceptualization, Methodology, Writing – original draft. **Markus Iyus Supiandi:** Writing – review & editing. **Thomas Joni Verawanto Aristo:** Project administration, Resources. **Ursula Dwi Oktaviani:** Data curation. **Dinn Wahyudin:** Supervision. **Asep Herry Hernawan:** Validation. **Anastasia Selvi:** Investigation. **Fiony:** Investigation. **Mawardi:** Investigation.

## Data Availability

Data for publication on Indigenous Knowledge Dalaynese research on Data in Brief (Original data) (Mendeley Data).
